# The coral core microbiome identifies rare bacterial taxa as ubiquitous endosymbionts

**DOI:** 10.1038/ismej.2015.39

**Published:** 2015-04-17

**Authors:** Tracy D Ainsworth, Lutz Krause, Thomas Bridge, Gergely Torda, Jean-Baptise Raina, Martha Zakrzewski, Ruth D Gates, Jacqueline L Padilla-Gamiño, Heather L Spalding, Celia Smith, Erika S Woolsey, David G Bourne, Pim Bongaerts, Ove Hoegh-Guldberg, William Leggat

**Affiliations:** 1 ARC Centre of Excellence for Coral Reef Studies, James Cook University, Townsville, Queensland, Australia; 2 QIMR Berghofer Medical Research Institute, Herston, Brisbane, Australia; 3 School of Pharmacy and Molecular Sciences, James Cook University, Townsville, Queensland, Australia; 4 Hawaii Institute for Marine Biology, University of Hawai‘i, Mānoa, HI, USA; 5 Department of Ecology, Evolution and Marine Biology, University of California, Santa Barbara, CA, USA; 6 Department of Botany, University of Hawai‘i, Mānoa, HI, USA; 7 Australian Institute for Marine Science, PMB 3, Townsville, Queensland, Australia; 8 The Global Change Institute, University of Queensland, Brisbane, Queensland, Australia

## Abstract

Despite being one of the simplest metazoans, corals harbor some of the most highly diverse and abundant microbial communities. Differentiating core, symbiotic bacteria from this diverse host-associated consortium is essential for characterizing the functional contributions of bacteria but has not been possible yet. Here we characterize the coral core microbiome and demonstrate clear phylogenetic and functional divisions between the micro-scale, niche habitats within the coral host. In doing so, we discover seven distinct bacterial phylotypes that are universal to the core microbiome of coral species, separated by thousands of kilometres of oceans. The two most abundant phylotypes are co-localized specifically with the corals' endosymbiotic algae and symbiont-containing host cells. These bacterial symbioses likely facilitate the success of the dinoflagellate endosymbiosis with corals in diverse environmental regimes.

## Introduction

Corals are found across a wide range of ocean habitats, from the warm sunlit tropical waters to the low light habitats of deep-water regions. The success of corals in these nutrient poor waters and across diverse environmental gradients relies on the endosymbiotic photosynthetic dinoflagellates that are abundant within the host's tissue layers. Recent estimates revealed there is also upwards of several thousand distinct bacterial phylotypes associated with individual coral colonies, resulting in one of the most diverse meta-organisms studied to date (Bayer *et* *al*., 2013). Bacterial symbioses are increasingly recognized as integral contributors to the acclimatization and adaptation of eukaryotes to environmental change (Bosch and McFall-Ngai, 2011; Sachs *et al.*, 2011; Bosch, 2013; McFall-Ngai *et al.*, 2013). Although these interactions have also been hypothesized to facilitate coral ecological successes, specific and obligate coral-bacterial symbioses have been difficult to distinguish within this complex system (Knowlton and Rohwer, 2003; Ainsworth *et al.*, 2010; Krediet *et al.*, 2013).

To accurately identify the role of bacterial communities and their contribution to their host, it is paramount to determine where the prokaryotic interactions occur and what their functional roles are (Ainsworth *et al.*, 2010; Fierer *et al.*, 2012; Bulgarelli *et al.*, 2013). In many cases, including algal–bacteria and squid–vibrio symbioses, it is often the phylotypes of low abundance in the surrounding environment (or members of the rare biosphere; Sogin *et al.*, 2006) that form the most physiologically significant (symbiotic) interactions (Decelle, 2013). This is particularly relevant for understanding the role of bacterial symbioses in diverse, complex and highly connected systems, such as reef corals. Furthermore, corals are exposed on a daily basis to substantial biotic and abiotic fluxes, such as re-suspension of marine sediments, fluctuations in the surrounding seawater chemistry, some of which are driven by endosymbiotic photosynthesis, and temperature (Ainsworth *et al.*, 2010). All of these factors have the capacity to substantially impact the host–microbial community interaction. The interface zone between the coral and surrounding environment is a thick and heavily colonized surface mucus layer, which is composed of photosynthetically fixed waste carbon. This layer is analogous to the soil environment of a plant root and forms a diffusion gradient within which microbes both utilize waste and alter the biochemical properties of the host-provided mucus. Beneath this outer mucus layer, other microhabitats provide niches for microbial interactions, such as the gut, the skeleton (which is open to the seawater and sediment environment) and the interface between these zones and the coral tissues. Although the coral–microbe–environment interface is a multifaceted and complex system supporting several thousand distinct bacterial phylotypes, the spatial arrangement, consistency and functional contributions of these interactions across individuals and reef ecosystems have yet to be addressed. Determining the structure of the coral microbial biosphere is paramount for identifying members of the coral whole community that form stable, consistent, symbiotic bacterial interactions with coral. Here we couple laser microdissection and next-generation sequencing to differentiate the coral microbial community and selectively sequence the microbiota associated exclusively with the coral tissues (symbiotic; namely the endosymbionts and episymbionts of the coral tissues) and the coral endodermal cells (endosymbionts), excluding skeletal, mucus, surface and loosely associated microbes.

The differentiation of the core microbiome of a host meta-organism, host microhabitat or environmental niche is a means to differentiate the stable and consistent associations from the whole community (Bäckhed *et al.*, 2012; Shade and Handelsman, 2012). This approach has been applied, for example, to differentiate the host–microbe interactions of the mammalian gut (and similar niche mammalian habitats) and in environmental settings, such as that of the plant root (Shade and Handelsman, 2012; Shafquat *et al.*, 2014). The identification of core members of the meta-organism subsequently allows for the differentiation of core pathways and metabolic functions that are provided by the host–microbe interaction (Shafquat *et al.*, 2014). However, the differentiation of a coral core microbiome, and core bacterial contributions to the coral host, has yet to be conducted. Here we determine the coral core microbiome by generating, and combining, the core microbiome within the coral whole community and coral niche habitats (that is, the coral holobiont or whole coral community, the symbiotic tissue community and the endosymbiotic community). We also predictively estimate the functional contribution of the coral core-associated bacteria (Langille, *et al.*, 2013). Utilizing this approach of coupling laser microdissection, fluorescence *in situ* hybridization (FISH) and next-generation sequencing, we can therefore accurately identify the spatial structure of coral-associated bacterial communities and determine their contribution to the meta-organism function. We further determine stable and consistent symbioses of coral hosts across geographically and ecologically distinct reef habitats by investigating coral species from both the Great Barrier Reef and Hawaiian Archipelagos. Analogous to the tightly coupled interactions of bacteria-plant rhizosphere (Lundberg *et al.*, 2012) and symbiotic oceanic algae (Thompson *et al.*, 2012), stable bacteria–dinoflagellate–coral associations likely provide corals with access to otherwise unavailable nutrients and metabolic pathways.

## Methods

### Sample collection and preparation

Corals, of the species *Acropora granulosa*, were collected from depths of 1–10 m, 20–30 m and 40–45 m from offshore reefs of the Northern Coral Sea, Australia. Coral samples were collected from five sites, including Mantis Reef, Lagoon Reef, Tydeman Reef, Yonge Reef and Ribbon Reef 5. Following collection, replicate coral samples were immediately stored in 100% molecular grade ethanol (*n*=3 per depth per site) or fixed with 4% paraformaldehyde (12 h at 4 °C) (FF). Following fixation, samples were washed in phosphate-buffered saline (PBS) and held in the cool until storage at 4 °C prior to processing. Ethanol-preserved coral branches were crushed and homogenized under liquid nitrogen and stored at −80 °C. A 40-mg aliquot of crushed and homogenized sample was collected from each sample and DNA was extracted using the MioBio Plant DNA Extraction kit (MoBio, Carlsbad, CA, USA; Cat. no. 12888) following a modification of the method described by Sunigawa *et al.* (2010). In brief, samples were digested in Proteinase K (50 mg ml^−1^) at 65 °C for 10 min and further homogenized in a Fastprep at 4.5 m s^−1^ for 2 min, following which the samples were processed as per the manufacturer's protocol. Following DNA extraction, samples were held at −20 °C prior to PCR amplification. Fixed (FF) coral branches were washed in PBS and then decalcified in a series of 20% DNA/RNA-free EDTA washes at 4 °C over a 2-week period, prior to paraffin embedding (PE) and sectioning at 7 μm. FFPE samples were used for tissue sampling from replicate embedded tissue branches, laser microdissection and for localization using FISH. Sampling of the symbiotic bacterial community (tissue-associated community) was conducted by collection using DNA/RNAse-free stainless steel biopsy cores (2 mm diameter) of coral polyps from decalcified, washed coral tissues. The cores from the FFPE samples were collected, dewaxed in xylene (reagent grade) and washed in ethanol (molecular grade) and DNAse/RNAse-free water prior to DNA extraction using the MoBio Plant DNA Kit (as described above). Sectioned tissues were cleared in a series of washes with reagent grade xylene, molecular grade ethanol and DNA/RNA-free water, prior to dissection of cell clusters (200 × 20 μm^2^) from the epithelial and closely associated gastrodermal tissue layers of replicate samples using a Zeiss PALM Microbeam microscope (Hamburg, Germany). Cell clusters were collected directly onto the lid of 0.2-ml tube and immediately capped and stored at −20 °C prior to DNA extraction. Laser capture microdissection-dissected coral clusters were stored in 0.2-ml tubes at −20 ^o^, and DNA extraction was conducted using the QIAamp DNA Micro Kit (as per the manufacturer's instructions) (Hilden, Germany; Cat. no. 56304).

*Montipora capitata* samples (*n*=16) were collected by technical divers using closed circuit rebreathers (Inspiration Rebreather, Silent Diving LLC, Brockville, ON, Canada) at 56-m depth (2011). All samples were collected from the middle of a dense *M. capitata* reef located offshore of Ma‘alaea Harbor, west Maui (20°48.2473' N, 156 °39.6050' W) and placed in separate plastic bags. Each sample was haphazardly selected and separated by at least 3 m distance (as measured with fin kicks). Sample bags were placed in a darkened, covered bucket with ambient seawater and processed within 4 h in a darkened laboratory on board the R/V *Ka‘imikai-O-Kanaloa*. Each sample was photographed and immediately frozen at −80 °C. *Leptoseris* spp. samples (*n*=29) were collected by the *Pisces V* manned submersible at three *Leptoseris* reefs offshore of Olowalu, west Maui at 65–75 m depths (20°48.65' N, 156°42.98' W; dive P5-757), 96–99 m depths (20°46.32' N, 150°40.15' W; dive P5-755) and 121–123 m depths (20°45.94' N, 156°40.20' W; dive P5-755) in 2011. At each site, representative corals ~20–30 cm in diameter were haphazardly selected from the middle of a *Leptoseris* reef, with each collected sample separated by at least 10 m in distance. A small, triangular piece of coral spanning from the middle to the outer edge of the coral head was removed using a Schilling Titan 4 manipulator (Houston, TX, USA) arm and placed in an individual sample container in the sampling basket. Collected samples were kept in a darkened container with ambient seawater and *in situ* temperatures and processed in a darkened laboratory onboard the R/V *Ka‘imikai-O-Kanaloa* within 4 h of ascent to the surface. Each sample was photographed, sampled for DNA and physiological analyses and then immediately frozen at −80 °C.

Coral branches of each species were crushed and homogenized under liquid nitrogen and stored at −80 °C. Habitat sampling from replicate branches was conducted, whereby coral polyps and coral skeleton-associated tissues were dissected from the coral branch from replicate coral branches of each species and also crushed under liquid nitrogen (as above). A 40-mg aliquot of crushed and homogenized sample was collected from each sample and DNA extracted using the MioBio Plant DNA Extraction Kit following a modification of the method described by Sunigawa *et al.* (2010). In brief, samples were digested in Proteinase K (50 mg ml^−1^) at 65 °C for 10 min and further homogenized in a Fastprep at 4.5 m s^−1^ for 2 min, following which the samples were processed as per the manufacturer's protocol. Following DNA extraction, samples were held at −20 °C prior to PCR amplification.

### PCR amplification, 454 tag sequencing and sequence analyses

Bacterial 16 S rRNA genes were PCR amplified from genomic template DNA in a single-step 30 cycle PCR (HotStarTaq Plus Master Mix Kit, Qiagen, Valencia, CA, USA) under the following conditions: 94 °C for 3 min, followed by 28 cycles of 94 °C for 30 s; 53 °C for 40 s and 72 °C for 1 min; after which a final elongation step at 72 °C for 5 min, using bacteria-specific barcoded primers 27 F/519 R with 454 A adaptor sequence. Following PCR, all amplicon products from different samples were mixed in equal concentrations and purified using Agencourt Ampure beads (Agencourt Bioscience Corporation, Beverly, MA, USA). Samples were sequenced utilizing Roche 454 FLX titanium instruments and reagents (MR DNA, Shallowater, TX, USA) and negative controls for each amplification and sequencing stage were utilized. DNA isolation, amplification and sequencing was conducted separately for each coral species and tissue type (August 2012 undertaken at James Cook University, November and December 2012 undertaken at the Hawaii Institute for Marine Biology). DNA extraction utilized previously unopened extraction kits for each set of samples from each species and preparation type. Negative controls of elute solution were utilized at all amplification and sequencing stages. The sequence data were processed using both a proprietary analysis pipeline (www.mrdnalab.com, MR DNA) and the software package QIIME where sequences were depleted of barcodes and primers and then short sequences <200 bp were removed, sequences with ambiguous base calls and sequences with homopolymer runs exceeding 6 bp were removed. Sequences were then denoised and chimeras removed and operational taxonomic units (OTUs) were defined at 97% similarity using QIIME (Caporaso *et al.*, 2010). OTUs were then taxonomically classified using BLASTn against the curated GreenGenes database (DeSantis *et al.*, 2006), and the core microbiomes were determined. Dendograms were generated using QIIME and visualized using the Interactive Tree of Life software (ITOL.embl.de)(Letunic and Bork, 2007). The statistical analysis and data mining was conducted using the Calypso software (http://bioinfo.qimr.edu.au/calypso/). Significantly different abundant OTUs were identified by Poisson regression (glm in R using family=quasipoisson and F-test to identify significance of model parameters). Differences in community diversity (richness and Shannon index) were identified by analysis of variance and regression analysis (modeling diversity as dependent variable and tissue and depth as explanatory variables). Significant differences in the global bacterial community composition were identified by analysis of similarity (Bray–Curtis distance), ADONIS (including depth, tissue type, and interaction of depth and tissue as explanatory variables) and RDA (also including depth and tissue as explanatory variables as listed above). For functional metagenome prediction, PICRUST (Langille *et al.*, 2013) was applied using GreenGenes database (v13_5) as reference. The functional profiles were subjected to the Calypso software for statistical analysis. PCoA was calculated based on KEGG KO functions and differences in KEGG Pathways were assessed by the analysis of variance test.

### The core microbiome

The core microbiome of each coral species was identified using QIIME and was determined by plotting OTU abundance in the core at 2% intervals (from 0% to 100% of samples). Phylotypes not consistently present in at least 30% of samples, of each preparation type, were considered to represent the individual variability of colonies. In the current study, a phylotype presence in at least 30% of samples was chosen as a conservative representation of the core microbiome. In selecting a 30% representation in the current study as the minimum representation, we determined the minimum percentage of samples at which net change in core OTU abundance from the previous 2% was found to be zero (that is, a rate of change of zero, was found to occur at 30% representation). A 30% core microbiome was found to be the lowest percentage at which core OTU abundance was stable across core microbiomes (Supplementary Figure S2). The coral core microbiome at 50% sample representation and 75% sample representation were also annotated and recorded. Phylotype conservation at 50% and 75% are consistent with previous research on core microbiome annotations. Therefore we also refer to the 50% coral coral microbiome and 75% coral core microbiome.

### Fluorescence *in situ* hybridization

Branches of *A. granulosa* were fixed individually in 50 ml of 4% paraformaldehyde (Electron Microscopy Services, Hatfield, PA, USA, Catalogue no. 15710) in DNase/RNase-free PBS (pH 7.4) (distilled water DNAse/RNAse-free GIBCO 500 ml Catalogue no. 10977-10, Roche 10 × 4 l Catalogue no. 11666789001). Following fixation, samples were washed in DNase/RNase-free PBS solution and stored in 50% ethanol:DNase/RNAse-free PBS solution. The coral samples were decalcified in DNase/RNase-free 20% EDTA at 4 °C with constant rocking and processed (washes of 50% EtOH, 70% EtOH, 95% EtOH (3times), 100% EtOH (3times), xylene (3times)) and infiltration with molten paraffin (3times), prior to embedding in molten paraffin. PE coral samples were sectioned at 7 μm, collected onto microscope slides (Menzel, Braunschweig, Germany; Cat. no. SF41296SP), dried overnight at 37 ºC and stored at −20 ºC. The tissue sections were dewaxed by washing three times in fresh 100% xylene (5 min per wash), and rehydrated by three washes in 100% EtOH (5 min per wash), prior to standard FISH protocol (Roller *et al.*, 1994; Ainsworth *et al.*, 2006). The bacterial probes used were each labeled with Cy3 flourochrome and include the EUB338mix general bacterial probes (GCTGCCTCCCGTAGGAGT, GCAGCCACCCGTAGGTGT, GCTGCCACCCGTAGGTGT) (Daims *et al.*, 1999), non-EUB probe (ACATCCTACGGGAGGC) (Roller *et al.,* 1994), Actinobacteria probe (TATAGTTACCACCGCCGT) with the competitor probe (Roller *et al.,* 1994), (TATAGTTACGGCCGCCGT) and the Ralstonia probe (Sanguin *et al.*, 2006) (TCCTATAGCATGAGGCCTTG). Hybridization conditions and reagent concentrations were utilized as previously described (Roller *et al.,* 1994; Daims *et al.*, 1999; Ainsworth *et al.*, 2006), and all FISH experiments were run in parallel with non-EUB and no probe controls to allow visualization of background fluorescence.

## Results and discussion

### The coral biosphere

**Figure 1:**
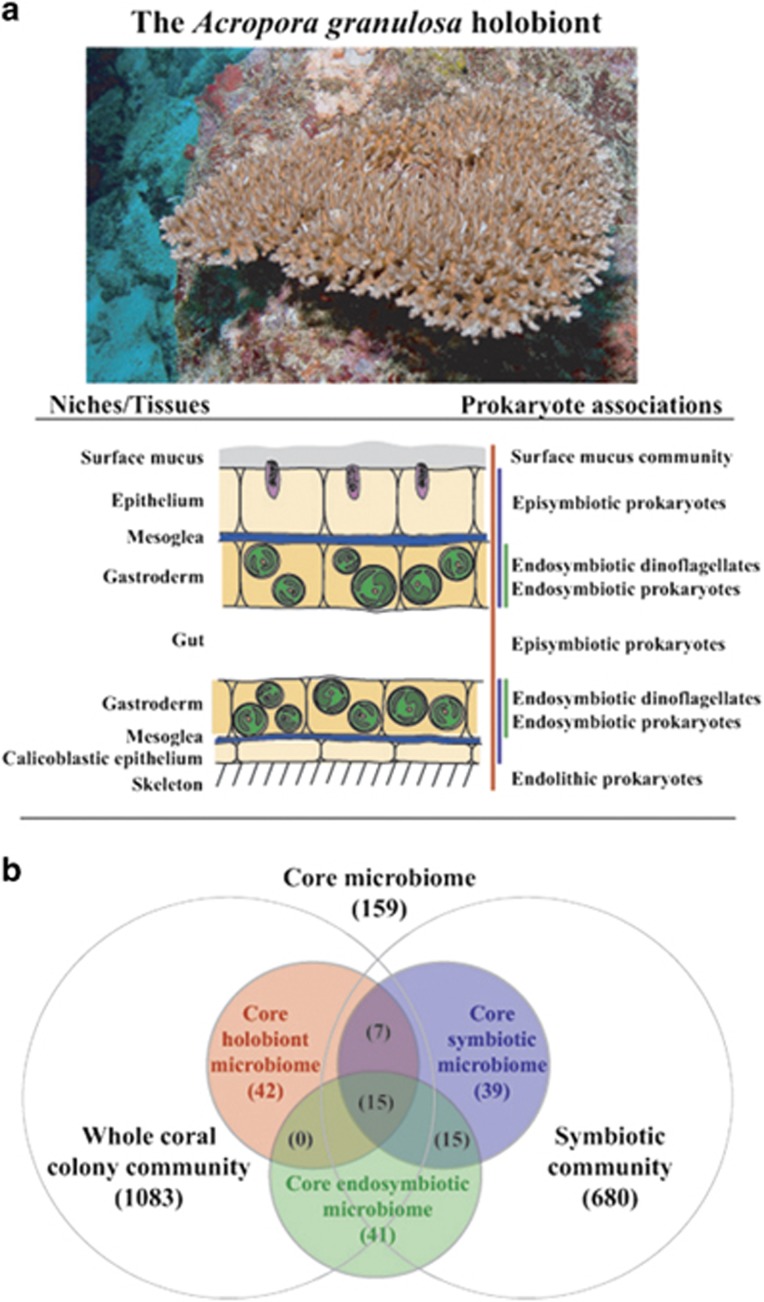
The host coral *Acropora granulosa* (pictured), the tissues layers of the host coral and regions of bacterial association (**a**) and Venn diagram of the inclusion of niche habitat associations within the coral microbiome (**b**). Coral holobiont is indicated in red, coral symbiotic tissue community is indicated in blue and coral endosymbiotic community is indicated in green.

**Figure 2:**
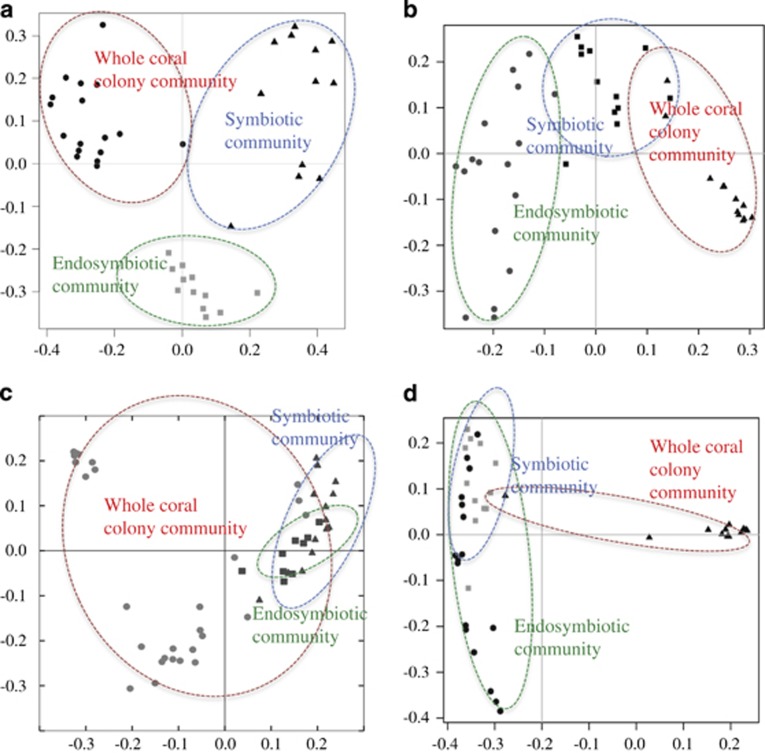
PCoA (Bray–Curtis) plot of bacterial associations within coral host habitats of the coral *Acropora granulosa* (**a**). PCoA (Bray–Curtis) plot of predicted metagenomes within the whole coral colony community (**b**). PCoA (Bray–Curtis) plot of bacterial associations within coral host habitats of the core microbiome of the coral *A. granulosa* (**c**). PCoA (Bray–Curtis) plot of predicted metagenomes within the microbiome of the coral *A. granulosa* (**d**).

Bacterial DNA was isolated from the coral holobiont community (whole coral-associated community), niche microhabitats of host tissues (cored and dissected polyp tissues removed from the skeleton and washed and dissected skeletal-associated tissue) and cells that host the endosymbiotic dinoflagellate (isolated using laser capture microdissection (Figure 1)). Bacterial 16S rRNA genes were amplified, sequenced by 454 pyrosequencing and analyzed bioinformatically (Caporaso *et al.*, 2010). In total, 326 055 sequence reads were obtained from *A. granulosa* (collected from the Coral Sea, Australia, from 4 to 40 m depths, *n*=33), 403 563 sequence reads were returned from *Leptoseris* spp. (collected from 65 to 125 m depths off Maui, Hawai‘i, USA, *n*=28) and 131 844 sequences reads from *Montipora capitata* (56 m depth off Maui, Hawai‘i, USA, *n*=16). Significant differences were evident between the holobiont (whole coral colony community), tissue symbiotic community (comprising endosymbiotic and episymbiotic tissue regions) and endosymbiotic bacterial communities in each of the coral species (Figure 2). These differences included indices for community structure (*P*=0.01 ADONIS, *P*=0.0005 RDA), diversity (Shannon diversity, *P*=0.0001) and richness (*P*=0.02) in the coral *A. granulosa* (Figure 2, Supplementary Figure S2, Supplementary Table S1).

### The coral core microbiome

**Figure 3:**
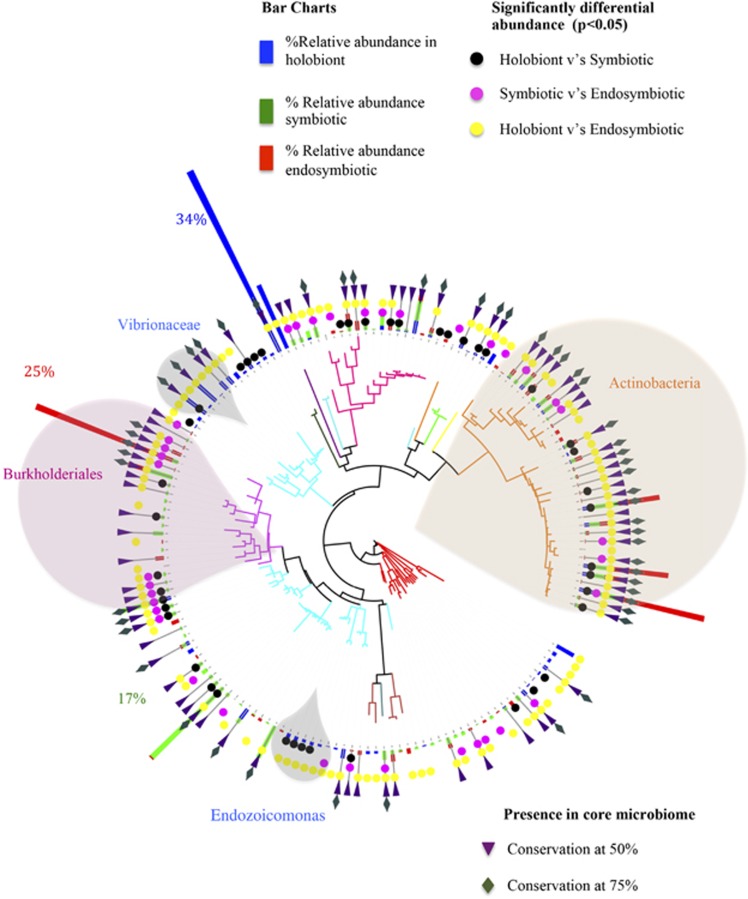
Dendrogram (Tree of Life) of coral-associated bacteria demonstrating average relative abundance of coral-associated bacterial communities within specific tissue regions of *Acropora granulosa* from the Great Barrier Reef and Coral Sea. Blue abundance bars represent holobiont (whole community) core microbiome, green abundance bars represent the specific members of the endosymbiotic and episymbiotic core microbiome of the coral tissues (tissue associated) and red abundance bars represent the specific members of the core microbiome from endosymbiotic regions. Individual bacterial phylotypes found to have significantly differential relative abundance (*P*<0.05) between the holobiont and symbiotic community are shown in black, holobiont and endosymbiotic community are shown in yellow and symbiotic and endosymbtioic community are shown in pink. Bacterial classes are shown by color. 50% core annotation is represented by purple triangle; 75% core annotation is represented by green diamond.

**Figure 4:**
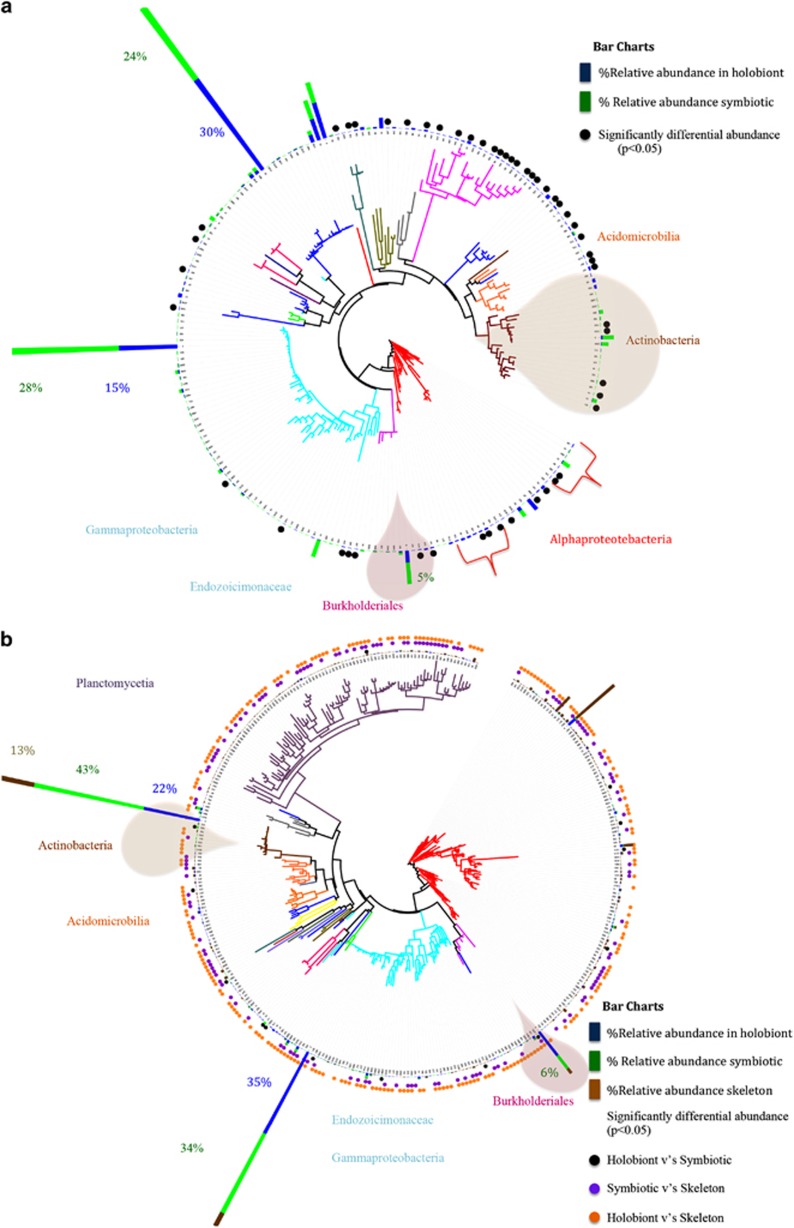
Dendrogram (Tree of Life) of coral-associated bacteria demonstrating average relative abundance of coral-associated bacterial communities within specific tissue regions of *Montipora capitata* (**a**) and *Leptoseris* spp. (**b**) from Hawai‘i. Blue abundance bars represent core holobiont (whole community) microbiome, green abundance bars represent the specific members within the endosymbiotic and episymbiotic core microbiome of the coral tissues and brown bars represent the specific member within the core microbiome of skeleton-associated (endolithic) microbiome. Individual bacterial phylotypes found to have significantly different relative abundance between the holobiont and symbiotic community are shown in black, holobiont and skeletal community are shown in brown and symbiotic and skeletal community are shown in purple. Bacterial classes are shown by color.

Of the total 1508 bacterial phylotypes identified in the whole community of *A. granulosa*, 159 phylotypes, representing 18 bacterial families, were identified as uniformly represented across a minimum of 30% all samples and used as a baseline from which to differentiate a potential coral core microbiome (Figure 3). In differentiating the coral core microbiome, we also combined the core microbiome determined in each preparation type separately (that is, the whole community, the symbiotic tissue community and the endosymbtiotic community). This was used as a means to account for the under-representation of significant, but rare or undetectable, community members within the whole community. For example, in *A. granulosa* (Figure 1b) 41 and 39 bacterial phylotypes were identified in both the core endosymbiotic and core symbiotic microbiomes, respectively, that were undetected in the core holobiont microbiome. Those phylotypes consistently found in at least 30% of samples in *A. granulosa* included a total of 64 phylotypes identified from the holobiont microbiome, 76 phylotypes identified from the symbiotic tissue microbiome and 71 phylotypes identified from endosymbiotic microbiome (Figure 1). A total of only 15 phylotypes were present within all three microbiomes, but 41 phylotypes were exclusively found in the endosymbiotic microbiome. The microbiome of the mesophotic corals *Leptoseris* spp. (Figure 4a) and *Montipora capitata* (Figure 4b) were identified to consist of a maximum of 204 phylotypes (out of 1424 bacterial phylotypes) and 350 phylotypes (out of 1433), respectively. Notably, in each species bacterial phylotypes were found to differ significantly in relative abundance within the habitat types sampled (*P*<0.05, Poisson regression; Figures 3 and 4). Most of the phylotypes that were identified in the coral microbiome with representation in at least 30% of samples were present in extremely low relative abundance or were rare within only the whole coral colony community. Of the 159 phylotypes of *A. granulosa* found within at least 30% of samples, 146 had <1% relative abundance and only 2 had a relative abundance >5% within the whole community. Similarly, in *Leptoseris* spp. and *M. capitata* only three and four phylotypes, respectively, were found to have >5% relative abundance in the whole community analysis (Figures 4a and b). This is a significant consideration and highlights the need for caution when determining coral bacterial interactions as core, stable or symbiotic, based solely on a high relative abundance within the hosts' whole community. To overcome reliance on relative abundance as an indication of importance, we further determine the bacterial taxa that are evident in a 50% core microbiome and 75% core microbiome analysis (Figures 3 4a and b).

Analysis of the core microbiomes of the coral *A. granulosa* demonstrate that only 0.09% of all bacterial phylotypes identified (holobiont or whole community) were in fact present in 90% of coral hosts'. We further show that only 0.5% were present in 75% of samples, 2.3% in 50% and only 5.5% of all the bacteria that were present in the coral hosts' whole community are present consistently in 30% of samples. As such, the vast majority of bacterial phylotypes that were amplified from individual corals were not in fact associated with all host coral colonies. This represents a change in how we understand the coral bacterial community and determine those members that are potentially important or symbiotic. In defining the core microbiome of *Arabidopsis thaliana* (Philippot *et al.*, 2013), the authors highlight the need for caution in assigning parameters for inclusion in the core microbiome. The authors annotate phylotypes to the core based on their presence in 50% of samples, in contrast to other systems where representation in 100% of samples is used to define the core. Shade and Handelsman (2012) further state that the environment, sequencing coverage and host of each system need to be considered when defining the core microbiome. We therefore first determine the representation of microbes in 30% of samples, and then annotate the coral core microbes evident in 75% of samples.

### Phylogenetic association within coral microhabitats

We further show clear spatial, phylogenetic associations evident within the coral core microbiome. These same phylogenetic and spatial distinctions in the microbial association with coral tissues were evident in all three mesophotic corals (Figures 3 and 4). We find that the *Alphaproteobacteria* orders *Rhizobiales* and *Caulobacterales* were only associated with the symbiotic microbiome but are not detectable in the whole coral colony community. Similarly, all members of the class Burkholderiales (16 bacterial phylotypes) (Figures 3 and 4, pink shaded regions) were only detectable in the symbiotic microbiome. These groups include bacteria that form symbiotic associations with plants, cyanobacteria, algae and marine invertebrates (Gruber-Vodicka *et al.*, 2011; Russell *et al.*, 2014). Conversely, all coral-associated members of the orders *Rickettisales* and *Rhodobacterales* (42 phylotypes) were exclusively amplified from the whole coral microbiome and not detected in the symbiotic microbiome. Significantly, all members of the order *Endozoicimonaceae*, which have previously been considered as important and dominant members of the coral microbiome (Lee *et al.*, 2012; Bayer *et al.,* 2013), were found exclusively in the whole organism microbiome and not in the symbiotic core microbiome. These phylogenetic associations are particularly evident in core microbiome analyses at 50% and 75% representation. It is likely that those phylotypes exclusively identified from the holobiont microbiome are localized to either the outer coral surface mucus layer or the coral skeleton, as they were not captured in the sequencing of isolated coral tissues. Phylotypes restricted to the outer interface regions (and not found in association with the tissues) are only likely to be functionally significant within these regions and are unlikely to directly impact coral physiology. However, these communities provide a first line of defence and protective role in the holobiont (Ritchie, 2006). Likewise, members of the *Vibrionaceae*, which are thought to be commensal and opportunistic coral pathogens, were not isolated from the symbiotic or endosymbiotic communities and are likely to only associate with the surface mucus in healthy coral (Figure 3).

### Universal symbioses

Finally, we identified bacterial phylotypes common to the coral core microbiome of species collected from widely separated geographical habitats. Seven phylotypes were found to be universally present in the coral core microbiome of all three mesophotic corals (*A. granulosa* (Coral Sea), *Leptoseris* spp. (Hawai‘i) and *M. capitata* (Hawai‘i). These individual corals span not only a wide depth range (5–130 m) but also a broad geographic area (7000 km) and provide evidence for specific coral–bacteria symbioses that are geographically stable and likely obligate within specific niche microhabitats of the host.

**Figure 5:**
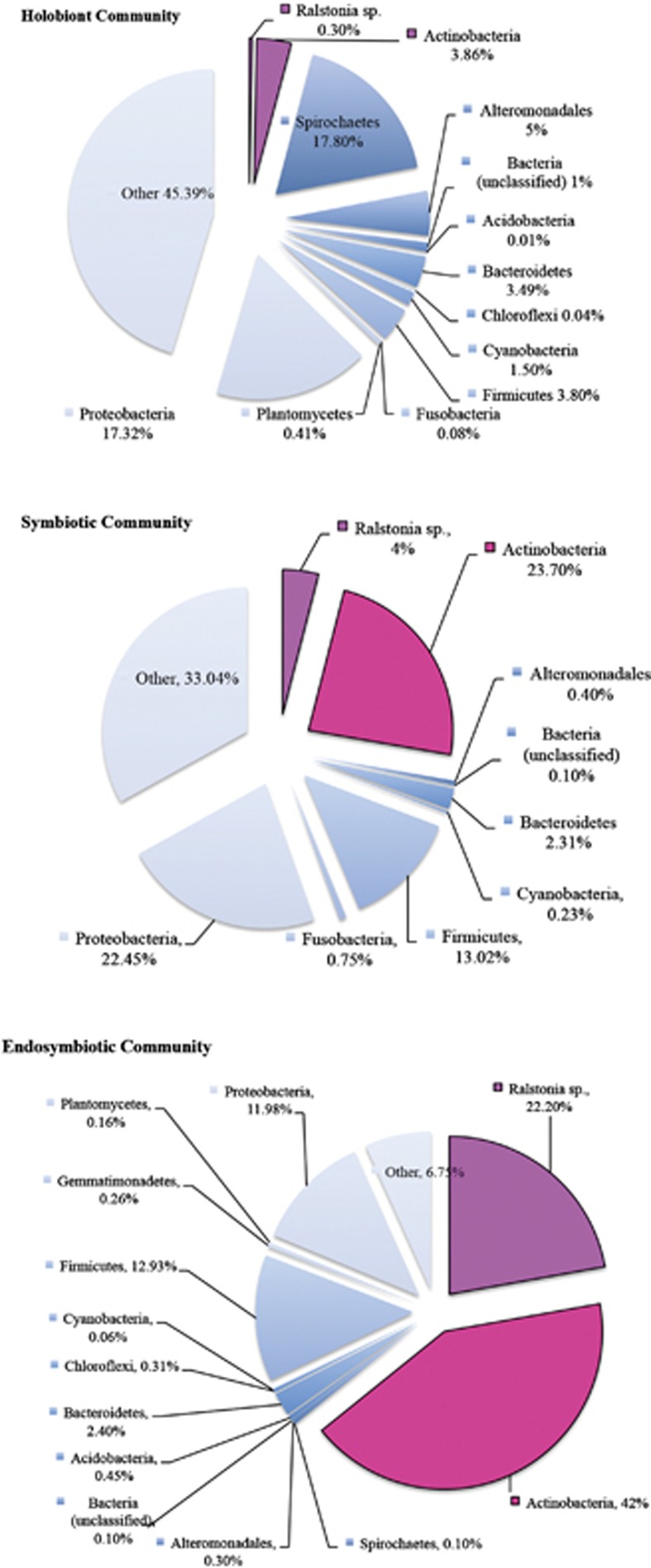
The percentage of relative abundance of groupings within endosymbiotic core microbiome, endosymbiotic and episymbiotic core microbiome and holobiont core microbiome.

**Figure 6:**
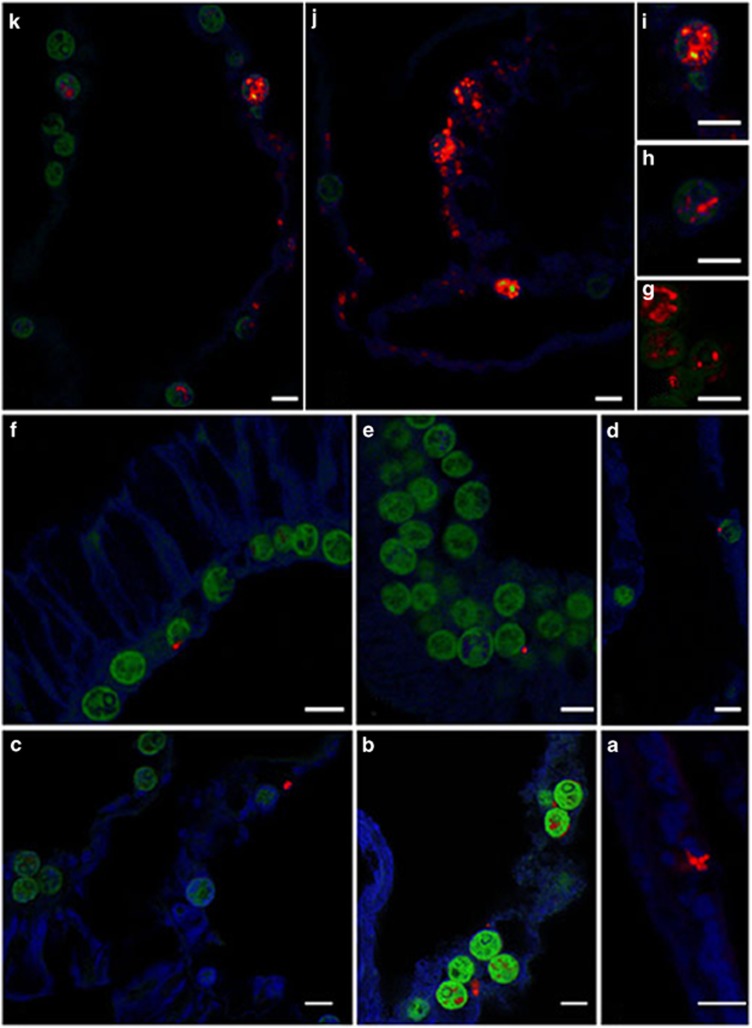
Localization of *Actinobacter* (**a**–**e**) and *Ralstonia* sp. (**f**–**k**), within the endosymbiotic dinoflagellates and gastrodermal cells of the coral host using FISH. Red indicates binding of the fluorescently labeled probe to bacteria cells within the host and endosymbiotic cells. Green indicates endosymbiotic dinoflagellates, blue indicates coral host tissues. Scale bar=10 μm.

The two most abundant types, *Actinomycelates* (99% sequence similarity to *Propionibacterium* sp.; accession number KM099464) and *Burkholderiales* (100% sequence similarity with *Ralstonia* sp.; accession number LN681565.1), were ubiquitously found in specific micro-niches in all coral species. These intracellular symbionts were present in high relative abundance within the endosymbiotic community (42% and 22%, respectively) but in low relative abundance within the coral whole community microbiome (~0.3% and 4%, respectively) (Figure 5). The localization of these conserved taxa within the coral host was determined using taxa- and group-specific probes in FISH. These two dominant bacterial types were subsequently localized as intracellular within the photosynthetic, endosymbiotic dinoflagellates (*Actinomycelates*, Figures 6a–e) and the coral host cells containing the dinoflagellates (*Ralstonia*, Figures 6f–k) using FISH. This is the first time that intracellular bacteria have been co-localized with endosymbiotic dinoflagellates in corals.

Meta-analysis of the published literature further reveals that these coral endosymbiotic genera are also found associated with 15 coral species collected globally (Table 1). Studies of the coral microbiome have not routinely reported bacterial phylotypes with relative abundances within the holobiont community lower than 2% or 5%, but when complete data sets are available, these phylotypes are predominantly rare in the whole community and undetectable in the adjacent seawater (Lee *et al.*, 2012; Sunagawa *et al.*, 2010). It is highly likely that these rare biosphere members are overlooked due to their low abundance in community-based studies, and as such their significance and contribution to the host organism has not previously been considered. These two key bacterial phylotypes are known to form symbiotic associations in other photosynthetic systems, where their functional roles include nitrogen-fixation, phytohormone production or promoters of plant growth (Hayat *et al.*, 2010; Bulgarelli *et al.*, 2013; Rastogi *et al.*, 2013; Sellstedt and Richau, 2013). As such, determining the functional contribution of these phylotypes to the host within their environmental niche is crucial to understanding their role in the coral meta-organism.

### Predicted roles of coral symbiotic bacteria

Bacterial symbiosis, through inter-kingdom interactions, might be a driver of ecological success and has been hypothesized as ecologically and evolutionarily significant. Interactions across phyla have substantial roles in organismal biology, influence ecosystem structure and may impact an organism's function and response to environmental changes (Robinson *et al.*, 2010; Caporaso *et al.*, 2011; Gonzalez *et al.*, 2011; Fierer *et al.*, 2012; Cleland, 2013). This is also likely to be the case for coral–bacterial interactions associated with the coral–dinoflagellate symbiosis. In highly complex samples, such as coral, that contain millions of cells from multiple eukaryotic sources and thousands of bacterial phylotypes, the isolation of target bacterial cells and their genomic information is technologically limited (Rinke *et al.*, 2014). Therefore, predictive estimates of microbial function can provide otherwise unobtainable insights into microbial function. Predictive metagenomic analysis (PICRUST; Langille *et al.*, 2013) was used to estimate the functional roles of bacteria within all preparations in both the coral communities and the generated core microbiomes (Supplementary Table S2). This analysis revealed that ABC transporters, sugar transporters and permeases were 3 of the 11 most abundant prokaryote genes (Table 2) and were significantly higher in abundance in the endosymbiotic core microbiome compared with the holobiont core microbiome (*P*<0.05) (Supplementary Figures S3 and S4). Ion couple and general transporters were also significantly enriched in the endosymbiotic core microbiome (*P*<0.05), indicating that significant metabolic exchange occurs between the coral host and endosymbiotic bacteria. *Ralstonia* sp. and *Propionibacterium* sp. account for between 65% and 75% of genes associated with transport (Supplementary Figure S5). In contrast, those related to amino-acid metabolism are accounted for by *Ralstonia* sp. (53%) with the next highest abundance being unassigned genera (19%). DNA repair, purine and pyrimidine metabolism proteins were also enriched in the endosymbiotic core microbiome (*P*<0.05), suggesting higher levels of DNA damage to endosymbiotic bacteria (Supplementary Figures S3 and S4). This is likely the result of increased levels of reactive oxygen species from *Symbiodinium* photosynthesis and skeletal amplification of light and ultraviolet rays (Wangpraseurt *et al.*, 2012). Other key pathways linked to symbiosis, such as nitrogen fixation and metal ion metabolism, were also highly abundant within the endosymbiotic community and therefore indicative of conserved bacterial endosymbiosis within corals.

## Conclusions

Bacterial symbioses are increasingly recognized as integral contributors to eukaryote's ability to acclimate and adapt to environmental change (Bosch and McFall-Ngai, 2011; Sachs *et al.*, 2011; Bosch, 2013; McFall-Ngai *et al.*, 2013). Corals harbor highly diverse and abundant microbial communities, with previous estimates suggesting upwards of several thousand distinct phylotypes (Bayer *et al.*, 2013). However, the coral core microbiome consists of a sustainably lower bacterial diversity, comprising only several hundred distinct phylotypes. Our study further provides strong evidence for niche habitat partitioning of the bacterial community within the coral host, an observation comparable to mutualistic bacterial symbioses in more complex metazoans. This also demonstrates that studies focusing on an organism's bacterial community as a ‘whole', particularly those studies that remove or disregard the rare phylotypes, do not accurately represent the bacterial phylotypes likely to be the most functionally significant to their hosts.

Furthermore, while specific, stable and obligate symbiotic coral–bacterial interactions have previously been considered unlikely (Krediet *et al.*, 2013), comparison across coral species and regions shows that consistent, specific interactions do occur. Here we reveal several globally stable coral–bacterial interactions across highly diverse reef habitats and show these symbiotic bacterial interactions are enriched from the rare biosphere of the coral host, and are intimately associated with the dinoflagellate endosymbionts. Analogous to the tightly coupled interactions of bacterial–plant rhizosphere (Lundberg *et al.*, 2012) and symbiotic oceanic algae (Thompson *et al.*, 2012), these dinoflagellate–bacteria–coral associations potentially provide the coral with access to otherwise unavailable nutrients and metabolic pathways. Bacterial symbioses such as these likely facilitate the success of the dinoflagellate endosymbiosis with corals in diverse environmental regimes, such as those investigated in the current study which include corals from shallow light-filled reef habitats and deeper water, mesophotic reefs.

We are, however, only beginning to understand the role of these interactions in relatively understudied, complex and connected marine systems, such as coral reefs. Much of the research to date on the diversity, ecology and physiology of reef-building corals has been undertaken within shallow tropical environments, and from our understanding of these systems, it is accepted that coral acclimation is driven by the host and/or its interaction with endosymbiotic dinoflagellates. For example, in low-light habitats, where photosynthesis is insufficient to meet the host energy requirements, adaptation is proposed to be supported through one of the three physiological adaptations; (i) switching reliance to heterotrophic feeding over autotrophy, (ii) switching of dominant endosymbiotic dinoflagellates types, and/or (iii) alterations to the host physiology that subsequently allow the host to persist within the new light regime of diverse reef habitats (Lesser *et al.*, 2009; Cooper *et al.*, 2010). Yet, evidence to date suggests that these interactions are not in fact sufficient to explain the growth and energy budget of the coral host (Kahng *et al.*, 2012), indicating that other adaptations or mechanisms of ecological facilitation may also contribute to corals success in diverse habitats. Given the metabolic capabilities of the symbiotic bacteria identified here, their global association with corals, and their localization within and around the endosymbiotic dinoflagellates, it is likely that they fulfil a role in the physiology and energy requirements of the coral host.

## Figures and Tables

**Table 1:** Association of rare biosphere bacteria symbionts within corals worldwide from published literature (Bayer *et al.*, 2013; Lee *et al.*, 2012; Sunagawa *et al.*, 2010)

*Host species*	*Location (depth)*	*Bacteria (genus)*	*Relative abundance*	*Reference*
*Montastrea faveolata*	Carilbbean (<5.5 m)	*Ralstonia* sp.	0.05%	Sunagawa *et al.*, (2010)
*Montastrea franksi*	Caribbean (<5.5 m)	*Ralstonia* sp.	0.07%	Sunagawa *et al.*, (2010)
*Diploria strigosa*	Caribbean (<5.5 m)	*Ralstonia* sp.	0.02%	Sunagawa *et al.*, (2010)
*Acropora palmata*	Caribbean (<5.5 m)	*Ralstonia* sp.	0.07%	Sunagawa *et al.*, (2010)
*Acropora cervicornis*	Caribbean (<5.5 m)	*Ralstonia sp.*	0.06%	Sunagawa *et al.*, (2010)
*Porites asteroids*	Caribbean (<5.5 m)	*Ralstonia* sp.	0.03%	Sunagawa *et al.*, (2010)
*Gorgonia ventalina*	Caribbean (<5.5 m)	*Ralstonia* sp.	0.06%	Sunagawa *et al.*, (2010)
*Acropora granulosa*	Coral Sea (<6 m)	*Ralstonia* sp.	0%	Current study
*Pocillopora verrucosa*	Red Sea (8 m)	*Ralstonia* sp.	5.9%	Lee *et al.*, (2012)
*Sarcophyton* sp.	Red Sea (8 m)	*Ralstonia* sp.	9.1%	Lee *et al.*, (2012)
*Sarcophyton* sp.	Red Sea (8 m)	*Ralstonia* sp.	11.5%	Lee *et al.*, (2012)
*Astreopora myriophthalma*	Red Sea (15 m)	*Ralstonia* sp.	1.8%	Lee *et al.*, (2012)
*A. myriophthalma*	Red Sea (19 m)	*Ralstonia* sp.	29.4%	Lee *et al.*, (2012)
*A. myriophthalma*	Red Sea (19 m)	*Ralstonia* sp.	22.3%	Lee *et al.*, (2012)
*A. granulosa*	Coral Sea (20 m)	*Ralstonia* sp.	0%	Current study
*A. granulosa*	Coral Sea (40 m)	*Ralstonia* sp.	0.12%	Current study
*Montipora capitata*	Hawaii (56 m)	*Ralstonia* sp.		Current study
*Leptoserius hawaiiensis*	Hawaii (65 m)	*Ralstonia* sp.		Current study
*L. hawaiiensis*	Hawaii (100 m)	*Ralstonia* sp.		Current study
*L. hawaiiensis*	Hawaii (125 m)	*Ralstonia* sp.		Current study
*A. faveolata*	Caribbean (<5.5 m)	*Propionibacterium* sp.	0.04%	Lee *et al.*, (2012)
*M. franksi*	Caribbean (<5.5 m)	*Proprionibacterium* sp.	0.04%	Lee *et al.*, (2012)
*D. strigosa*	Caribbean (<5.5 m)	*Propionibacterium* sp.	0.02%	Lee *et al.*, (2012)
*A. palmata*	Caribbean (<5.5 m)	*Propionibacterium* sp.	0.01%	Lee *et al.*, (2012)
*A. cervicornis*	Caribbean (<5.5 m)	*Propionibacterium* sp.	0.01%	Lee *et al.*, (2012)
*P. asterides*	Caribbean (<5.5 m)	*Propionibacterium* sp.	0.001%	Lee *et al.*, (2012)
*G. ventalina*	Caribbean (<5.5 m)	*Propionibacterium* sp.	0.02%	Lee *et al.*, (2012)
*A. granulosa*	Coral Sea (<6 m)	*Propionibacterium* sp.	1.64%	Current study
*Pocillopora verrucosa*	Red Sea	*Propionibacterium* sp.	<1.2%	Lee *et al.*, (2012)
*Sarcophyton* sp.	Red Sea	*Proprionibacterium* sp.	<0.7%	Lee *et al.*, (2012)
*A. myriophthalma*	Red Sea	*Proprionibacterium* sp.	<1.0%	Lee *et al.*, (2012)
*A. granulosa*	Coral Sea (20 m)	*Propionibacterium* sp.	3.55%	Current study
*Eunicella cavolini*	Caribbean (24 m)	*Propionibacterium* sp.	0.23%	Bayer *et al.*, (2013)
*A. granulosa*	Coral Sea (40 m)	*Propionibacterium* sp.	2.15%	Current study
*M. capitata*	Hawaii (56 m)	*Propionibacterium* sp.		Current study
*L. hawaiiensis*	Hawaii (65 m)	*Propionibacterium* sp.		Current study
*L. hawaiiensis*	Hawaii (100 m)	*Proprionibacterium* sp.		Current study
*L. hawaiiensis*	Hawaii (125 m)	*Propionibacterium* sp.		Current study

**Table 2:** Predicted KEGG pathways with significantly different abundance (*P*<0.05) between the coral holobiont (whole community), symbiotic and endosymbiotic communities

*KEGG pathway*	*Endosymbiont enriched*	*Symbiotic enriched*	*Possible function in symbiosis*
ABC transporters	✓		Transport of substrates, such as ions, sugars and lipids.
Amino-acid-related enzymes	✓	✓	Synthesis, degradation and utilization of amino acids
DNA repair and recombination proteins	✓		Repair of DNA
Other ion-coupled transporters	✓	✓	Ion transporters involved in environmental information processing
Oxidative phosphorylation	✓		Energy metabolism
Purine metabolism	✓		Nucleic acid metabolism
Pyrimidine metabolism	✓		Nucleic acid metabolism
Ribosome	✓		Translation
Transporters	✓		Transport of substrates, such as ions, sugars and lipids
Bacterial motility proteins		✓	Synthesis of flagellar
Function unknown		✓	Unknown
Secretion system		✓	Secretion of proteins, including those involved in nutrient uptake
