# Supplementary material for: The coral core microbiome identifies rare bacterial taxa as ubiquitous endosymbionts
Source: ISME J. 2015 Apr 17;9(10):2261–74. doi: 10.1038/ismej.2015.39 (PMC4579478; doi:10.1038/ismej.2015.39)
Supplement: Supplementary Information [file ismej201539x1.doc]

**Supplementary Figures**

**Supplementary Figure 1**. PCoA (Bray-Curtis) plot of bacterial associations within coral host habitats of the coral *Montipora capitata* (a) and *Leptoseris* spp. (b) aside, community diversity and richness.

**Supplementary Figure 2**. Plotted bacterial OTU abundance in core microbiome’s of 0 to 100% of samples, of the whole coral colony community (holobiont) (a) and endosymbiotic community (b) of the coral *A. granulosa.*

**Supplementary Figure 3**. PICRUSt determination of metagenomes and significantly different abundant (relative) KEGG pathways of the whole coral bacterial community of corals *A.granulosa* (a), *M. capitata* (b) and *Leptoseris* spp. (c) (ANOVA p<0.05)

**Supplementary Figure 4**. PICRUSt estimation of metagenomes and significantly different abundant (relative) KEGG pathways in the coral core microbiome of the corals *A.granulosa* (a), *M. capitata* (b) and *Leptoseris* spp. (c) (ANOVA p<0.05)

**Supplementary Figure 5.** PICRUSt estimation of metagenomes and annotation to bacterial phylotypes of the endosymbiotic core microbiome.

**Supplementary Tables**

**Supplementary Table 1.** Pairwise comparison from ANOVA for Shannon diversity, richness and ANOSIMS for *Acropora granulosa* (a) *Leptoseris sp.* (b) and *Montipora capitata* (c).

**Supplementary Table 2.** NSTI scores for predicted metagenomes of GBR and Hawaiian archipelago coral samples.
