# Supplementary figures and images for: The coral core microbiome identifies rare bacterial taxa as ubiquitous endosymbionts
Source: ISME J. 2015 Apr 17;9(10):2261–74. doi: 10.1038/ismej.2015.39 (PMC4579478; doi:10.1038/ismej.2015.39)

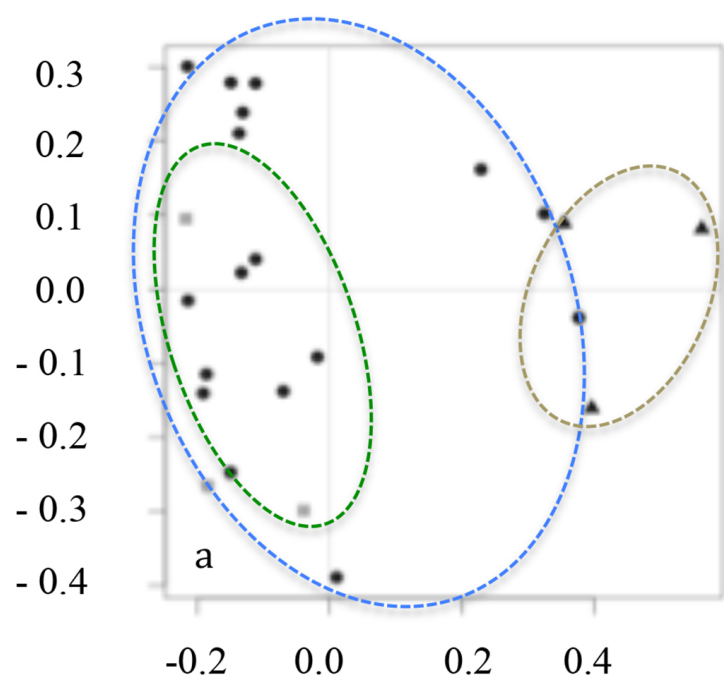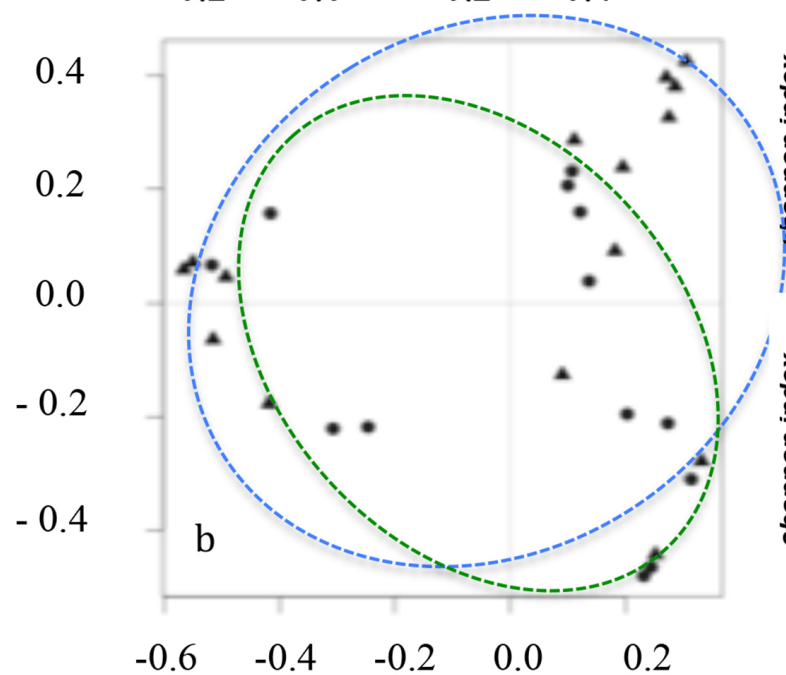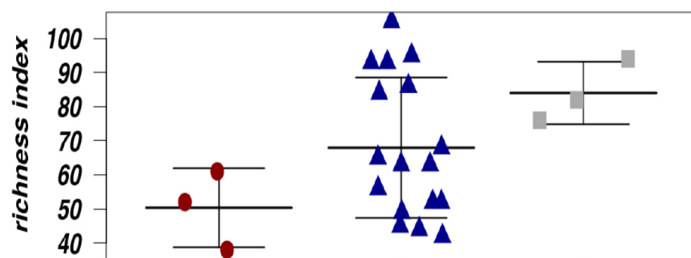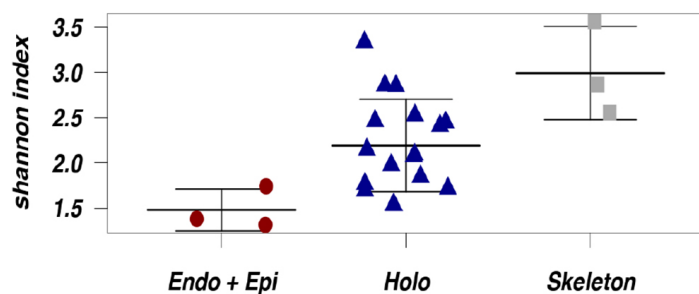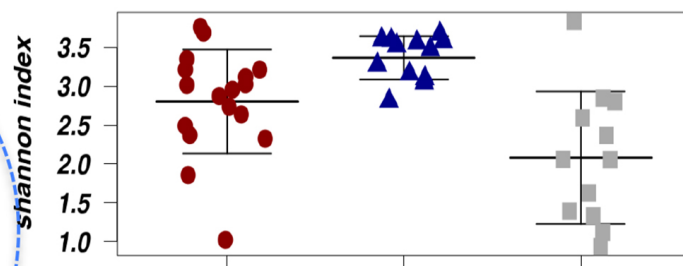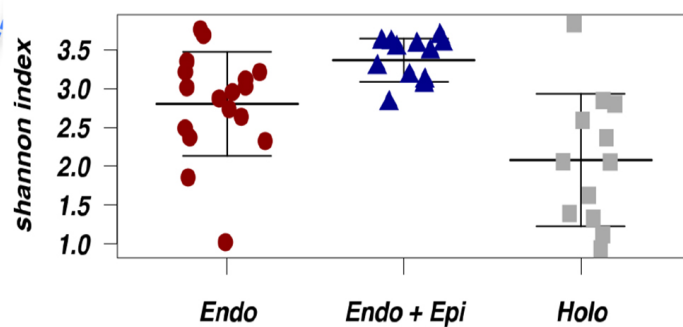

Supplement: Supplementary Figure 1 [file ismej201539x2.pdf]

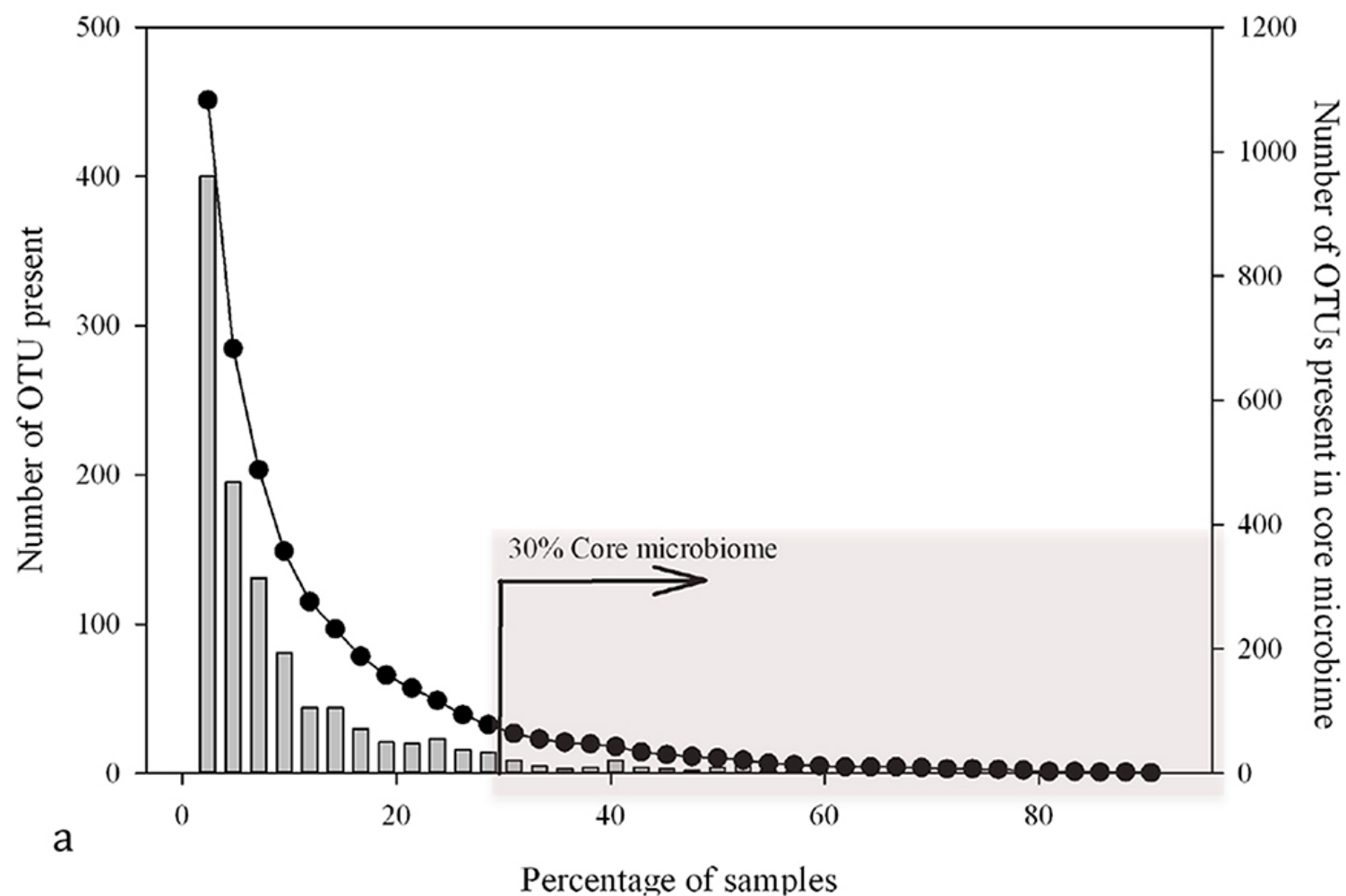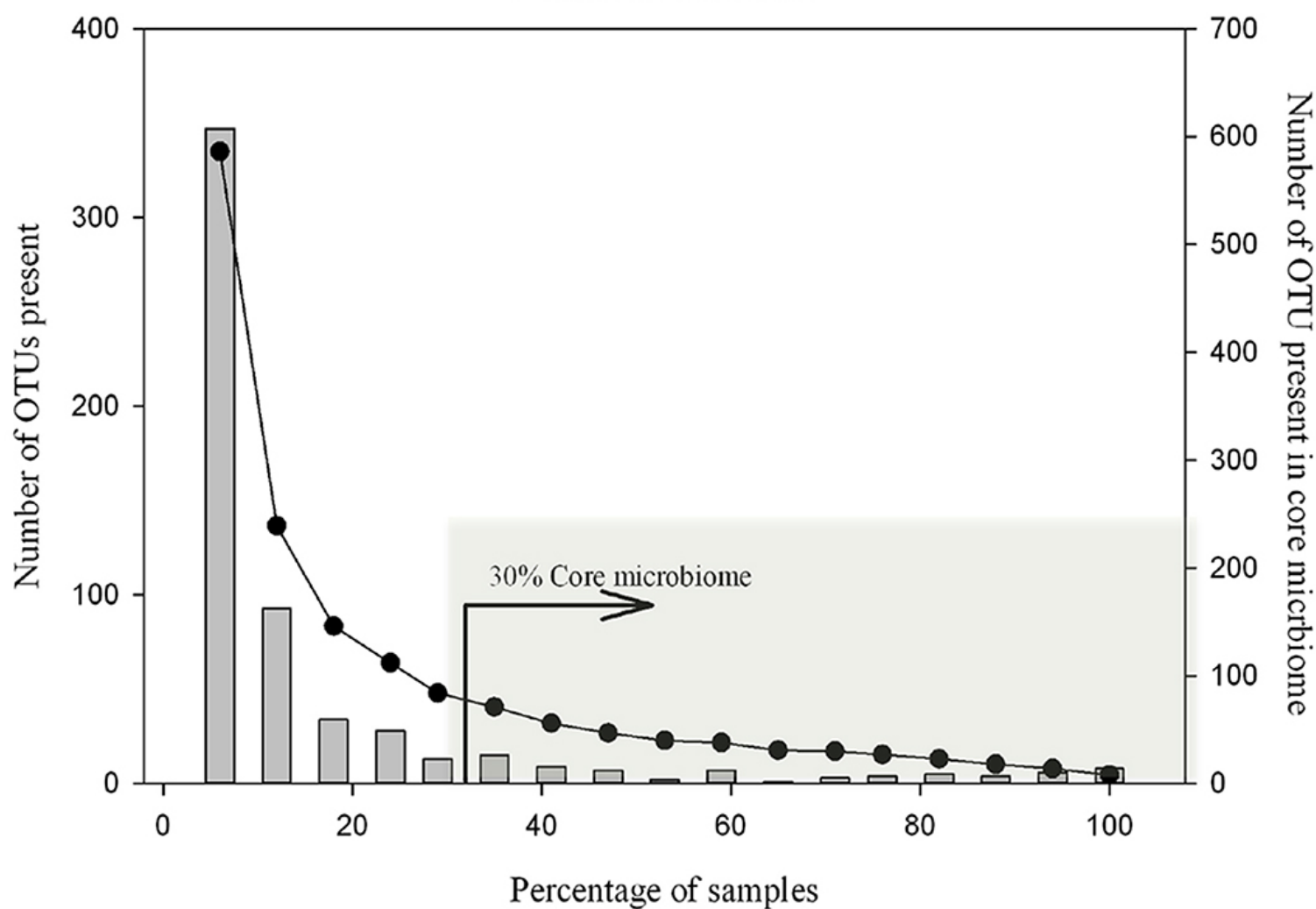

Supplement: Supplementary Figure 2 [file ismej201539x3.pdf]

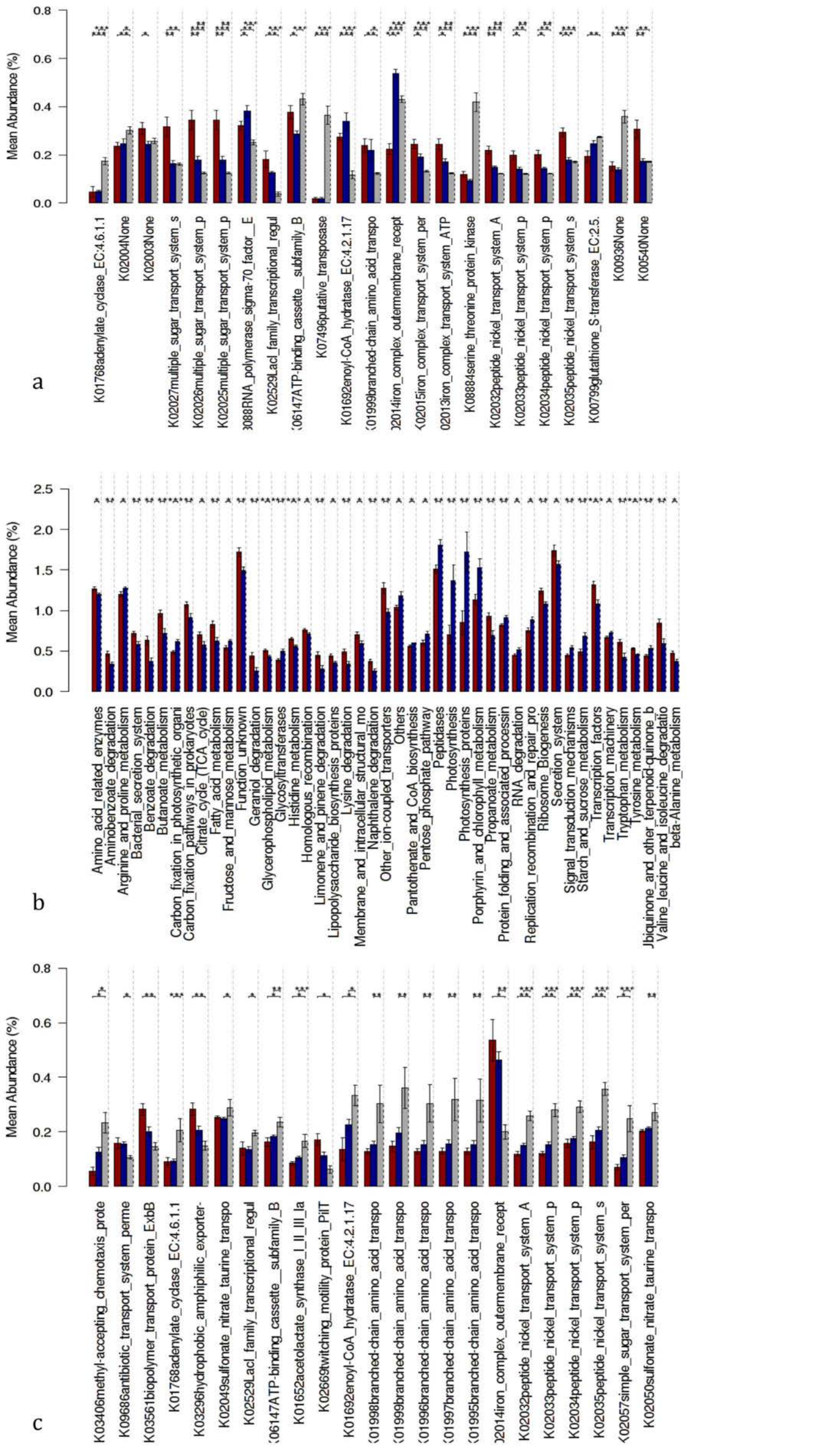

Supplement: Supplementary Figure 3 [file ismej201539x4.pdf]

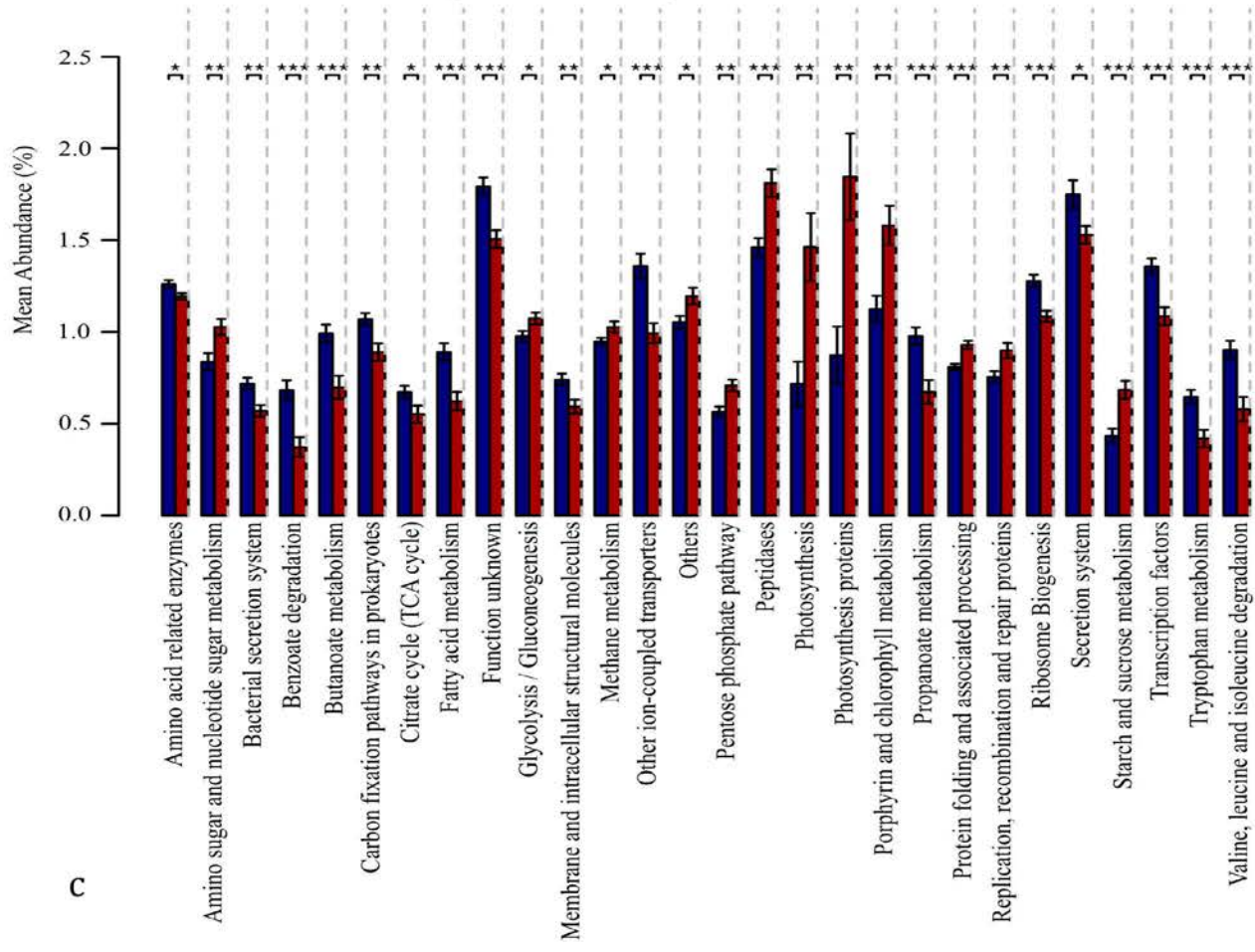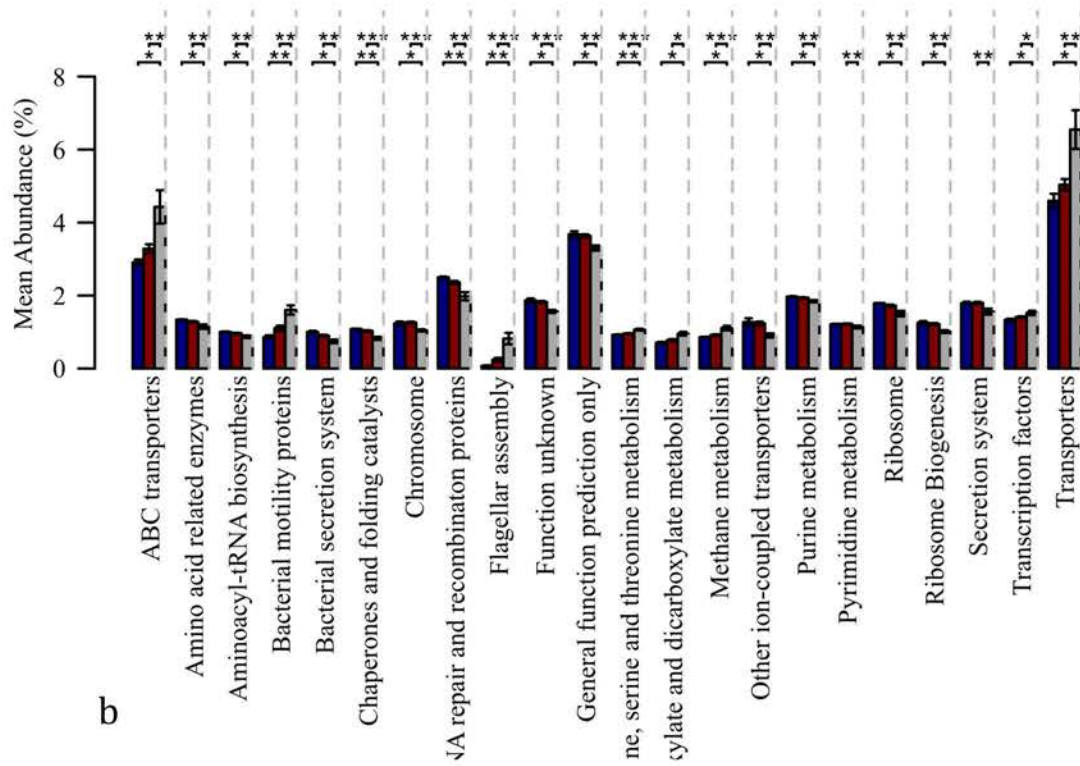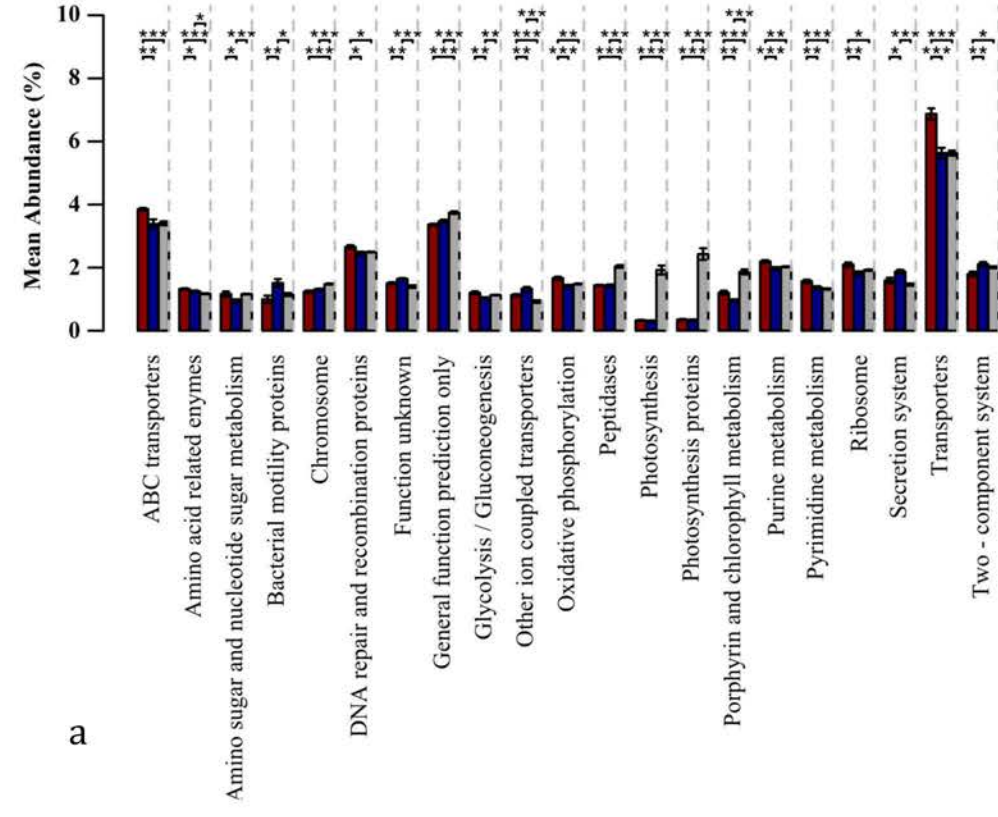

Supplement: Supplementary Figure 4 [file ismej201539x5.pdf]

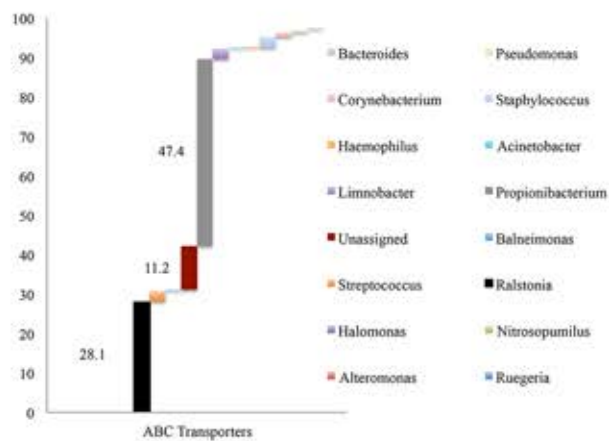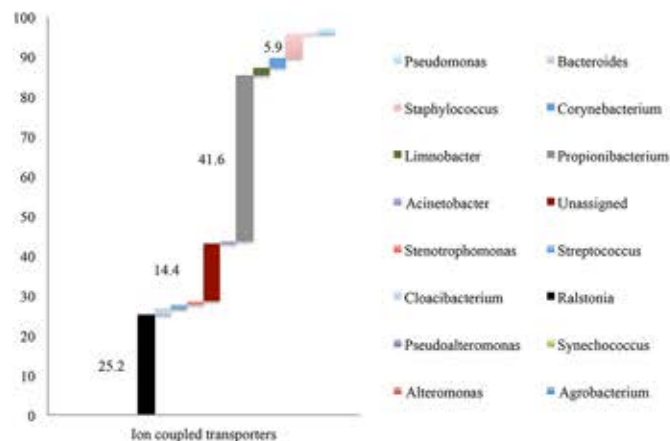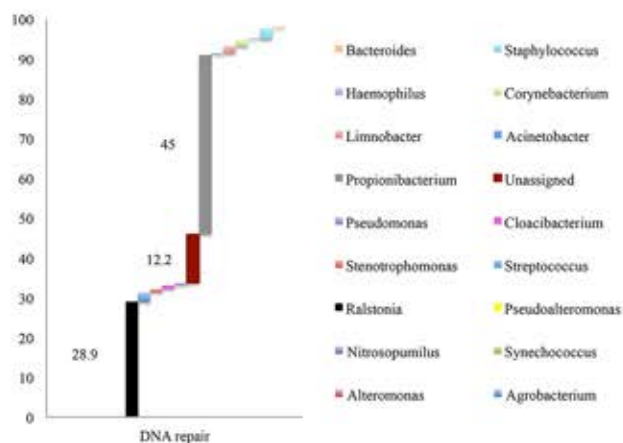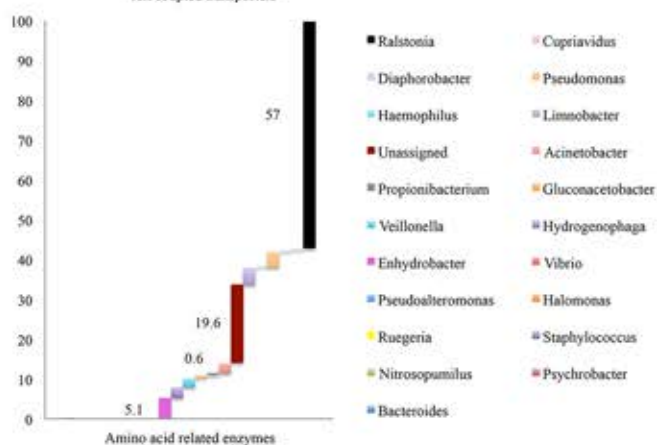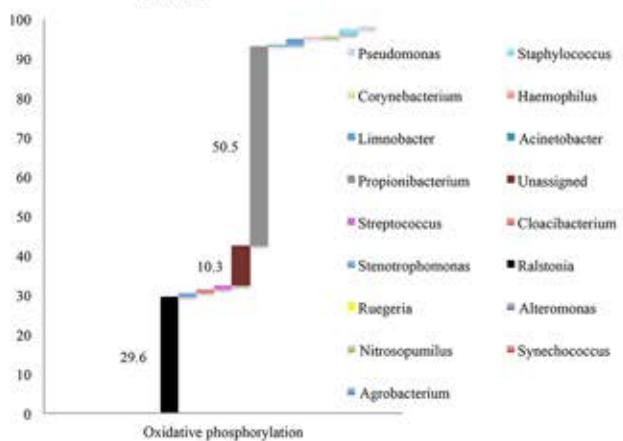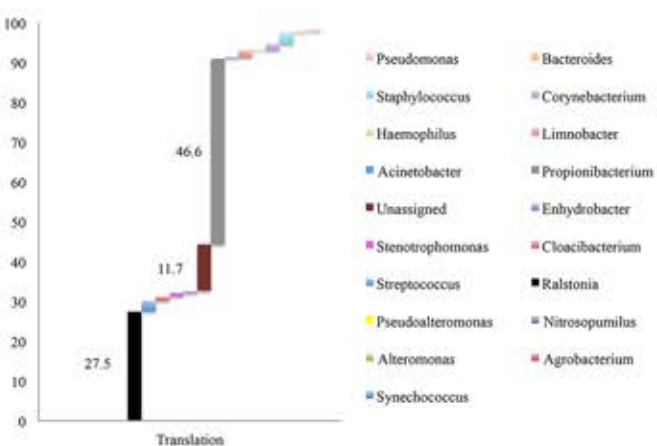

Supplement: Supplementary Figure 5 [file ismej201539x6.pdf]
