# Supplementary material for: The coral core microbiome identifies rare bacterial taxa as ubiquitous endosymbionts
Source: ISME J. 2015 Apr 17;9(10):2261–74. doi: 10.1038/ismej.2015.39 (PMC4579478; doi:10.1038/ismej.2015.39)
Supplement: Supplementary Table 1 [file ismej201539x7.pdf]

a		Symbiotic, p	Holobiont, p
Shannon diversity	Endosymbiotic	0.012	0.008
	Symbiotic		0.00005
Richness	Endosymbiotic	0.46	0.15
	Symbiotic		0.022
ANOSIMS	Endosymbiotic	0.001	0.001
	Symbiotic		0.001)
b			Holobiont
Shannon diversity	Symbiotic		0.028
Richness	Symbiotic		0.038
ADONIS	Symbiotic		0.035
c			Holobiont
Shannon diversity	Symbiotic		0.0045
Richness	Symbiotic		0.12
ADONIS	Symbiotic		0.003
