# Supplementary material for: The coral core microbiome identifies rare bacterial taxa as ubiquitous endosymbionts
Source: ISME J. 2015 Apr 17;9(10):2261–74. doi: 10.1038/ismej.2015.39 (PMC4579478; doi:10.1038/ismej.2015.39)
Supplement: Supplementary Table 2 [file ismej201539x8.pdf]

a) Great Barrier Reef	**Min.**	**Median**	**Mean**	**Max.**
Holobiont	0.08791	0.3311	0.3119	0.3692
Endosymbiotic	0.01725	0.02888	0.02928	0.04893
Symbiotic	0.0265	0.05791	0.05604	0.08057

b) Hawaii’	**Min.**	**Median**	**Mean**	**Max.**
Holobiont	0.05775	0.1072	0.1116	0.1794
Endsymbiotic	0.1087	0.1614	0.1485	0.1753
Skeleton associated	0.08822	0.1225	0.1177	0.1423
